# The Estimation of First-Phase Insulin Secretion by Using Components of the Metabolic Syndrome in a Chinese Population

**DOI:** 10.1155/2015/675245

**Published:** 2015-03-01

**Authors:** Jiunn-diann Lin, Chun-Hsien Hsu, Yao-Jen Liang, Wei-Cheng Lian, Chang-Hsun Hsieh, Chung-Ze Wu, Dee Pei, Yen-Lin Chen

**Affiliations:** ^1^Division of Endocrinology, Department of Internal Medicine, Shuang Ho Hospital, School of Medicine, College of Medicine, Taipei Medical University, Taipei 110, Taiwan; ^2^Department of Family Medicine, Cardinal Tien Hospital, School of Medicine, Fu-Jen Catholic University, New Taipei City 242, Taiwan; ^3^Department of Life-Science, Fu-Jen University, New Taipei City 242, Taiwan; ^4^Division of Endocrinology and Metabolism, Department of Internal Medicine, Buddhist Dalin Tzu Chi General Hospital, School of Medicine, Hualien 970, Taiwan; ^5^Division of Endocrinology and Metabolism, Department of Internal Medicine, Tri-Service General Hospital, Taipei 114, Taiwan; ^6^Division of Endocrinology and Metabolism, Department of Internal Medicine, Cardinal Tien Hospital, Medical School, Fu-Jen Catholic University, New Taipei City 242, Taiwan; ^7^Department of Pathology, Cardinal Tien Hospital, Medical School, Fu-Jen Catholic University, New Taipei City 242, Taiwan

## Abstract

*Aims*. There are two phases of insulin secretion, the first (FPIS) and second phase (SPIS). In this study, we built equations to predict FPIS with metabolic syndrome (MetS) components and fasting plasma insulin (FPI).* Methods*. Totally, 186 participants were enrolled. 75% of participants were randomly selected as the study group to build equations. The remaining 25% of participants were selected as the external validation group. All participants received a frequently sampled intravenous glucose tolerance test, and acute insulin response after the glucose load (AIRg) was obtained. The AIRg was considered as FPIS.* Results*. When MetS components were only used, the following equation was built: log (FPIS) = 1.477 − 0.119 × fasting plasma glucose (FPG) + 0.079 × body mass index (BMI) − 0.523 × high-density lipoprotein cholesterol (HDL-C). After FPI was added, the second equation was formulated: log (FPIS) = 1.532 − 0.127 × FPG + 0.059 × BMI - 0.511 × HDL-C + 0.375 × log (FPI), which provided a better accuracy than the first one.* Conclusions*. Using MetS components, the FPIS could be estimated accurately. After adding FPI into the equation, the predictive power increased further. We hope that these equations could be widely used in daily practice.

## 1. Introduction

Both deteriorated insulin sensitivity (S_I_) and impaired insulin secretion are recognized as 2 of the foremost forms of pathophysiology for type 2 diabetes (T2DM) [1, 2]. In the natural course of developing diabetes, the plasma glucose does not rise until the decompensation of the *β*-cell function to insulin resistance (IR) occurs. Moreover, the impaired *β*-cell function is a more critical factor than decreased S_I_ to determine the development of T2DM, especially in Asian people [3, 4].

Two phases of insulin secretion are widely known: the first-phase insulin secretion (FPIS) and the second-phase insulin secretion (SPIS). The FPIS is normally secreted by the *β*-cells within 10 min after being exposed to an acute rise in plasma glucose levels to reduce their emission [5], whereas the SPIS is the newly secreted insulin from *β*-cells after the FPIS. A better FPIS results in longer maintenance of normal glucose homeostasis before the occurrence of diabetes [5]. The FPIS is a sensitive indicator for the deterioration of insulin sensitivity [6, 7] because it decreases rapidly even in prediabetes stage (PreDM) [8]. Normally, it becomes completely disappeared when diabetes is diagnosed.

Numerous studies have documented the link between metabolic syndrome (MetS), the clustering of hyperglycemia, hypertension, obesity, and dyslipidemia, and an elevated risk of developing cardiovascular disease and diabetes [9, 10]. A consensus exists that IR is the core of MetS [11–14]. Simultaneously, each abnormal MetS component adversely affected insulin secretion independently [15]. Components such as the body mass index (BMI), triglyceride (TG), high-density lipoprotein cholesterol (HDL-C), and fasting plasma glucose (FPG) levels were all shown to be related to insulin secretion [11, 16]. Although, as mentioned, the FPIS is important, no readily available accurate method exists for measuring the first ISEC. In this study, we built an equation to estimate the FPIS by using routine clinical variables and MetS components. We hope that the estimated FPIS can be widely used in clinical settings.

## 2. Participants and Methods

### 2.1. Participants

In total, 186 participants were enrolled in this study. Either they were self-referred or health professionals had referred them for diabetes screening. They had no history of diabetes and, therefore, they took no diabetes medications at the time of the study. They were defined as having a normal glucose tolerance (NGT), PreDM, and T2DM according to the criteria published by the American Diabetes Association in 2012 [17]. Otherwise, none of the participants had a remarkable medical or surgical history. In total, 51 participants had NGT, 40 had PreDM, and 95 had T2DM. Before participating in the study, they were instructed by physicians and dietitians not to receive any medication known to affect glucose or lipid metabolism and to remain on a stable diet for at least 1 wk before the study. On the day of the first study, an entire routine workout was completed by participants to exclude those with significant cardiovascular, endocrine, renal, hepatic, and respiratory disorders. The study protocol had been approved by the hospital's institutional review board and ethics committee; all participants provided written informed consent prior to participating. To validate our equation, 75% of the participants were randomly selected. Based on these participants, an optimal equation was built for estimating the FPIS. This equation was subsequently used to calculate the FPIS among the remaining 25%, who constituted the external validation group.

### 2.2. Study Protocol

Frequently sampled intravenous glucose tolerance test (FSIGT): all tests were performed at the clinical research center. On the day of the study visit, after a 12 h overnight fast, one catheter was placed on both arms of each participant. A bolus of 10% glucose water (0.3 g/kg) was given. Another bolus of regular human insulin (Novo Nordisk Pharmaceutical, Princeton) 0.05 units/kg was injected 20 min after the glucose load. Blood samples for plasma glucose and insulin levels were collected at 0 min, 2 min, 4 min, 8 min, 19 min, 22 min, 30 min, 40 min, 50 min, 70 min, 100 min, and 180 min. The data were inputted into a Bergman minimal model [1], and then the S_I_, glucose effectiveness (GE), and acute insulin response after the glucose load (AIRg) were obtained. The AIRg was considered the FPIS, and the product of the S_I_ and the AIRg was the disposition index (DI).

The calculations of HOMA-IR and HOMA-*β* (homeostasis model assessment of insulin resistance and the *β*-cell function) were performed according to Matthew's equation [18].

Plasma was separated within 1 h of blood withdrawal and stored at −30°C until the analysis. Plasma glucose was measured using a glucose analyzer by employing an oxidase method (YSI Model 203, Scientific Division, Yellow Spring Instrument Company, Inc., Yellow Spring, OH, USA). Plasma insulin was assayed using a commercial solid phase radioimmunoassay technique (Coat-A-Count insulin kit, Diagnostic Products Corporation, Los Angeles, CA, USA) with intra- and interassay coefficients of variance of 3.3% and 2.5%, respectively. Serum TG was measured using the Fuji Dri-Chem 3000 analyzer (Fuji Photo Film Corporation, Minato-Ku, Tokyo, Japan) by employing the dry multilayer analytical slide method. The serum HDL-C concentration was determined using the enzymatic cholesterol assay method after dextran sulfate precipitation.

### 2.3. Statistical Analysis

The data were tested for normal distribution by using the Kolmogorov-Smirnov test and for the homogeneity of variances by using the Levene test. Continuous variables were expressed as mean ± standard deviation. Among the data, FPIS, FPI, S_I_, and DI were not normally distributed and were logarithmically transformed. An independent *t*-test was used to evaluate the demographic data, the clinical characteristics, and the parameters derived from the FSIGT between the 2 groups (a study and external validation group). To build the equation to estimate the FPIS, we used the stepwise method in multiple regression analysis. We adopted sex, age, and the MetS components as independent variables and the FPIS as the dependent variable. Although FPI is not a component of MetS, it was found to be strongly related to FPIS; another equation with the FPI as the independent variable was also built.

These equations were subsequently used to calculate the FPIS among the remaining 25% of participants. The correlation between the calculated FPIS and measured FPIS was measured using Pearson's *r* correlation coefficient. Higher correlation coefficients (*r*) indicate a superior prediction accuracy. Hierarchical multiple regression method was also used to examine predicting power between these equations.

All statistical analyses were performed using the SPSS software system, version 13.0 (SPSS Inc., Chicago, IL, USA). All *P* values less than 0.05 were considered statistically significant.

## 3. Results

In the study, 140 and 46 participants were classified into the study group and the external validation group, respectively.  Table 1 shows the demographic data, FPG and FPI, plasma lipids, and variables derived from the FSIGT of these two groups. There was no significant difference in these measurements between the two groups. The demographic data of NGT, PreDM, and T2DM are shown in Table 2. The participants in the T2DM group were older and had a higher FPG compared to the NGT group. Log (FPIS), log (DI), and log (GE) were significantly lower in the DM group.

To identify the parameters that contribute most to the FPIS, the correlations between the FPIS and different parameters were evaluated; the results are shown in Table 3. The FPIS was significantly correlated to age (*r* = −0.398, *P* = 0.000), BMI (*r* = 0.264, *P* = 0.002), FPG (*r* = −0.475, *P* = 0.000), HDL-C (*r* = −0.190, *P* = 0.034), and log (FPI) (*r* = 0.382, *P* = 0.000).

**Figure 1:**
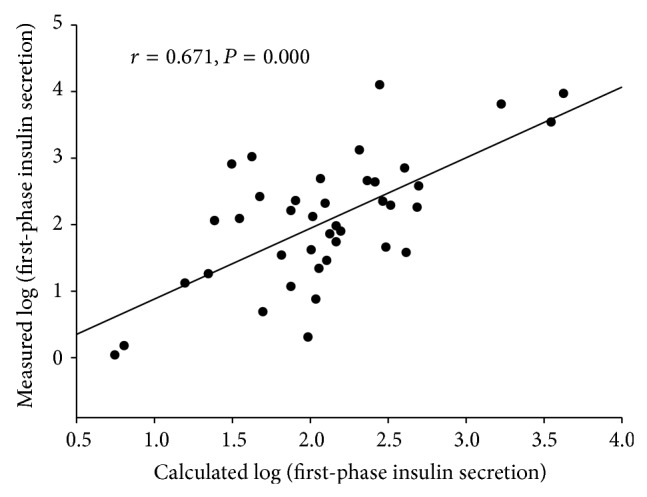
The correlation between the calculated first-phase insulin secretion and measured first-phase insulin secretion by using metabolic syndrome components in the external validation group.

Only MetS components were used in multiple linear regression analysis. Three of them were selected from regression analysis, and the equation was built and is shown as log (FPIS) = 1.477 − 0.119 × FPG + 0.079 × BMI − 0.523 × HDL-C (standard coefficients are shown in Table 4). Subsequently, as mentioned, this equation was used to calculate the FPIS of the external validation group. The correlation between calculated log (FPIS) and the measured log (FPIS) was assessed, and the results are shown in Figure 1. The *r* value was 0.671, and *P* was 0.000.

Because the FPI is also considered a surrogate for the FPIS, it was also added to multiple linear regression analysis to build a second equation and, unlike the first, 4 factors were selected, and the following equation was formulated: log (FPIS) = 1.532 − 0.127 × FPG + 0.059 × BMI − 0.511 × HDL-C + 0.375 × log (FPI). The difference of predicting power of FPIS between the first and the second equation was determined using hierarchical multiple regression method. The *r* ^2^ increased significantly in both of the study (*P* = 0.000) and external validation groups (*P* = 0.049) after adding log (FPI) into FPG, BMI, and HDL-C.

**Figure 2:**
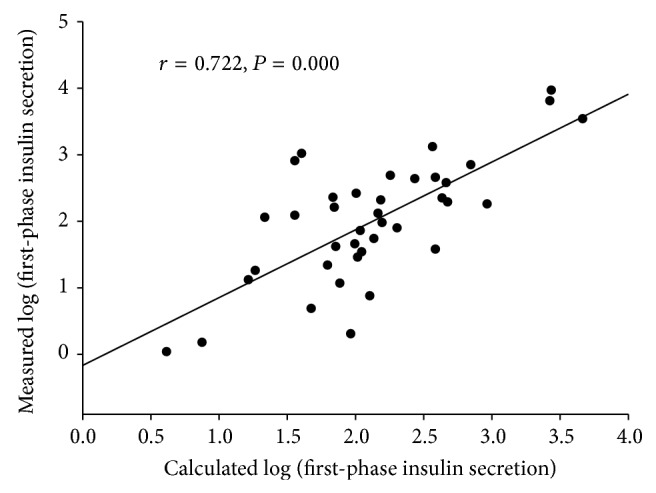
The correlation between the calculated first-phase insulin secretion and measured first-phase insulin secretion by using metabolic syndrome components and fasting plasma insulin in the external validation group.

The correlation between the calculated FPIS and the measured FPIS in the external group was also evaluated, and the results are shown in Figure 2. The calculated log (FPIS) determined the measured log (FPIS) with good accuracy in the external validation group (*r* = 0.722, *P* = 0.000). Compared to HOMA-*β*, both equations showed a better predictive accuracy for the FPIS (*r* = 0.551, *P* = 0.000). The standardized coefficient between the associated factors and the FPIS is shown in Table 4.

## 4. Discussion

In this study, we built an equation by using routine clinical measurements and MetS components to predict the FPIS in participants with different levels of glucose tolerance. Because of the tight correlation between FPI and FPIS, FPI was also added into analysis to build a second equation to improve predictive accuracy. To verify our results, external validation was also performed. Although previous studies have been done to predict the FPIS, most of them enrolled only nondiabetic participants [3, 11, 19–21]. Because we believe that our study results are informative and reliable, they could be applied widely to the public health domain and in clinical settings.

The FPIS is the immediately releasable stored insulin in *β*-cell granules [22]. The most widely used standardized methods for measuring the FPIS are the hyperglycemic clamp and the FSIGT. By using the hyperglycemic clamp, Chiu et al. reported that the combination of ethnicity and BMI could be used to predict 16.6% of the variance of the FPIS in NGT participants [21]. Similarly to this study, van Haeften et al. found that a combination of sex, BMI, and the family history of T2DM (FH) could predict the FPIS in the NGT and PreDM participants with similar accuracy (*r* ^2^ = 0.152, *P* < 0.0001 and 0.250, *P* < 0.0001, resp.) [19]. By using the FSIGT, Alford et al. showed the best prediction accuracy among these 3 studies (*r* ^2^ = 0.27). In our study, by using only the MetS component, *r* ^2^ was as elevated as 0.45, which is a substantially superior result compared to those of the mentioned studies. After adding the FPI into the model, *r* ^2^ could have been improved to 0.521 in the external validation group.

In our study, the FPG, BMI, HDL-C, and FPI were selected among all other factors and inputted into multiple linear regression analysis. Because the predominant function of *β*-cells is to maintain glucose in homeostasis, the FPG provides the most substantial contribution in the regression model [11]. Our results are in line with earlier studies which showed that the FPIS deteriorated as the FPG levels increased from NGT to diabetic range, which suggested that the FPG level is the most critical determinant for assessing the deterioration of *β*-cell function [7].

Following the FPG, the BMI was the second most critical factor inputted in the model. The results were not surprising because the evidence has shown that people with a higher BMI would have a better *β*-cell function because of the larger amount of *β*-cell mass [11, 19, 21, 23]. van Haeften et al. reported that the BMI is a critical contributor to the FPIS in the NGT and the PreDM participants (*r* ^2^ = 0.096 and 0.090, resp.). In agreement with their findings, our study also demonstrated that the BMI explained a similar level of variance for the FPIS in participants across the spectrum of glucose tolerance (*r* ^2^ = 0.107, data not shown). It could be questioned that waist circumference was not added into analysis in the study as it is the key component of MetS. However, waist circumference was not measured in the study, so we could not estimate FPIS using waist circumference. Evidence showed that BMI was highly correlated with waist circumference (*r* = 0.900 in men and *r* = 0.889 in women) in Chinese [24]. Moreover, Chiu et al. demonstrated that BMI is better marker than waist-hip ratio to predict first insulin secretion [21]. Therefore, BMI could replace the waist circumference to predict first insulin secretion.

Because the lower HDL-C is associated with IR [11, 16], we postulate that a negative correlation between HDL-C and the FPIS should exist. Both Hanley et al. and Gower et al. have supported this hypothesis [11, 16]. This study also produced similar findings (*r* = −0.190, *P* = 0.034). However, the Bardini study results demonstrated that there was no significant correlation between HDL-C and early-phase insulin secretion in NGT subjects but there was positive correlation between HDL-C and early-phase insulin secretion in impaired glucose tolerance subjects, which do not match ours [25]. Several explanations are available that could be used to resolve this dispute. First, ethnic differences might be the reason for the discrepancies between studies [16, 21]. Second, the FPIS was measured in the current study by using the FSIGT, which was identical with the Hanley and Gower studies. In addition, early-phase insulin secretion in the Bardini study was assessed using only a surrogate marker derived from the oral glucose tolerance test (OGTT), which is a less accurate method compared to the FSIGT. Third, in the Bardini study, the participants with T2DM were not enrolled. These differences could have a profound effect on the relationship between the FPIS and the HDL-C.

The FPI, which is not a routinely used measurement, is associated with *β*-cell function [7, 11, 18, 26]. Our results, which are compatible with the observations by Hanley et al., showed a significant association between the FPI level and the first ISEC (*r* = 0.361, *P* = 0.000). After combining the FPI and the MetS components into the equation, the predictive accuracy improved further (*r* ^2^ increase from 0.450 to 0.521 in the external validation group). This finding might also imply that the FPI and MetS could have affected the FPIS via different pathways because the improvement of *r* ^2^ is substantial. Further well-designed study is needed to address the issue.

The *β*-cell function declines as age increases, even in participants with NGT [21, 27]. Earlier study has shown that this negative influence of aging on the *β*-cell function might be attributed to the gradual loss of the abilities of both proinsulin converted to insulin and the decreased baseline proliferative activity of *β*-cell compared with younger adults [28, 29]. Moreover, evidence also showed that aging positively correlated with enhanced glucose-induced *β*-cell apoptosis in vitro [29]. In our study, we demonstrated that age was negatively correlated with the FPIS (*r* = −0.398, *P* = 0.000). However, age was not selected using multiple linear regression analysis. The finding might be attributed to the strong correlation between age and either FPG, BMI, or FPI which could reduce the impact of age on FPIS. In other words, the effect of age was masked by other stronger relationships between the FPIS and the MetS components.

To the best of our knowledge, the current study is the first to formulate an equation for estimating the FPIS by using the MetS components and the FPI level. However, our study has limitations. First, the body fat content and its distribution, which were known to be associated with IR and the *β*-cell function [30], were not measured in the study. Measuring these factors in the study might help further improve predictive power of the equation. Second, FH was not assessed in the study. It has been established that participants with an FH of T2DM have a reduced *β*-cell function and a decreased *β*-cell response to IR compared to those without [31]. Third, this study investigated only one ethnic group: the Han people. Thus, the application of our results to other ethnic groups should be exercised with caution. Finally, this study is only a cross-sectional study. In future studies, using a baseline that incorporates MetS components to estimate the FPIS would be more valuable. However, even with these limitations, we still believe that our finding could be easily and widely used in clinical settings.

In conclusion, by using the MetS components, the FPIS could be predicted with reliable accuracy (*r* = 0.671). After adding the FPI to the equation, the predictive power increases further (*r* = 0.722). These equations could be widely used in daily practice.

## Figures and Tables

**Table 1:** Demographic data of the study and external validation groups.

	Study group	Ext. val. group	*P* value
*n*	140	46	
Sex (male/female)	69/71	25/21	0.552
Age (y)	50.7 ± 13.5	50.8 ± 14.7	0.910
Body mass index (kg/m^2^)	25.2 ± 3.9	25.8 ± 5.1	0.366
Systolic blood pressure (mmHg)	121.5 ± 13.0	118.3 ± 16.2	0.186
Diastolic blood pressure (mmHg)	76.3 ± 8.0	73.4 ± 7.7	0.076
Triglyceride (mmol/L)	1.3 ± 0.6	1.5 ± 0.6	0.112
HDL-C (mmol/L)	1.1 ± 0.3	1.1 ± 0.4	0.350
Fasting plasma glucose (mmol/L)	7.8 ± 2.8	7.5 ± 3.0	0.540
Fasting plasma insulin (pmol/L)	30.5 (12.2–61.3)	27.6 (14.4–62.0)	0.353
First-phase insulin secretion (*μ*U/min)	115.0 (23.5–426.4)	114.9 (24.4–430.4)	0.822
Insulin sensitivity (10^−4^·min^−1^·pmol^−1^·L^−1^)	1.274 (0.5–3.4)	1.6 (0.3–3.3)	0.501
Disposition index	87.8 9 (20.3–900.8)	95.3 (17.0–408.7)	0.830
Glucose effectiveness (10^−2^·dL·min^−1^·kg^−1^)	0.016 ± 0.010	0.015 ± 0.010	0.314
HOMA-IR	1.7 (0.6–3.1)	1.7 (0.7–3.1)	0.615
HOMA-*β*	22.1 (6.9–83.0)	20.0 (8.5–92.0)	0.334

Data are expressed as mean ± SD or median (interquartile range). Ext. val. group: external validation group.

HDL-C: high-density lipoprotein cholesterol; HOMA-IR and HOMA-*β*: homeostasis model assessment of insulin resistance and *β*-cell function.

**Table 2:** Demographic data of normal glucose tolerance, prediabetes, and diabetes groups.

	Normal glucose tolerance	Prediabetes	Diabetes
*n*	51	40	95
Age (y)	42.5 ± 17.2^2,3^	54.4 ± 11.9^1^	53.7 ± 10.3^1^
Body mass index (kg/m^2^)	26.1 ± 5.9	24.9 ± 3.1	25.1 ± 3.5
Systolic blood pressure (mmHg)	118.1 ± 10.9	121.0 ± 14.7	121.9 ± 14.9
Diastolic blood pressure (mmHg)	74.0 ± 6.8	76.1 ± 8.5	76.4 ± 8.5
Triglyceride (mmol/L)	1.2 ± 0.6	1.4 ± 0.6	1.4 ± 0.6
HDL-C (mmol/L)	1.1 ± 0.3	1.1 ± 0.3	1.1 ± 0.3
Fasting plasma glucose (mmol/L)	4.6 ± 0.5^2,3^	6.4 ± 0.4^1,3^	9.9 ± 2.2^1,2^
Fasting plasma insulin (pmol/L)	49.5 (9.3–81.1)	25.5 (7.5–61.7)	23.0 (14.4–44.3)
First-phase insulin secretion (*μ*U/min)	517.5 (183.0–5144.7)^2,3^	123.6 (35.5–390.7)^1^	37.8 (11.6–158.3)^1^
Insulin sensitivity (10^−4^·min^−1^·pmol^−1^·L^−1^)	0.8 (0.2–3.2)	1.9 (0.6–4.4)	1.4 (0.6–2.9)
Disposition index	893.9 (240.0–2447.1)^2,3^	54.8 (21.7–894.6)^1^	40.7 (8.3–182.5)^1^
Glucose effectiveness (10^−2^·dL·min^−1^·kg^−1^)	0.020 ± 0.010^2,3^	0.014 ± 0.008^1^	0.014 ± 0.010^1^
HOMA-IR	1.7 (0.4–2.7)	1.3 (0.3–3.1)	1.7 (0.8–3.3)
HOMA-*β*	134.0 (27.9–352.4)^2,3^	29.0 (10.4–72.6)^1,3^	13.0 (6.2–26.4)^1,2^

Data are expressed as mean ± SD or median (interquartile range). HDL-C: high-density lipoprotein cholesterol; HOMA-IR and HOMA-*β*: homeostasis model assessment of insulin resistance and *β*-cell function.

^1^ *P* value < 0.05 when compared with “Normal glucose tolerance” group; ^2^ *P* value < 0.05 when compared with “Pre-diabetes” group; ^3^ *P* value < 0.05 when compared with “diabetes” group.

**Table 3:** Pearson correlation between the clinical parameters and log (first-phase insulin secretion) in the study group.

Variables	*r*	*P* value
Age	−0.398	0.000
Body mass index	0.264	0.002
Systolic blood pressure	−0.044	0.623
Diastolic blood pressure	0.030	0.740
Triglyceride	−0.064	0.463
HDL-C	−0.190	0.034
Fasting plasma glucose	−0.475	0.000
Log (FPI)	0.382	0.000
Log (insulin sensitivity)	−0.184	0.035
Log (HOMA-IR)	0.231	0.006
Log (HOMA-*β*)	0.551	0.000

HDL-C: high-density lipoprotein cholesterol; FPI: fasting plasma insulin; HOMA-IR and HOMA-*β*: homeostasis model assessment of insulin resistance and *β*-cell function.

**Table 4:** Multiple linear regression of the associated factors with log (first-phase insulin secretion) in the 2 equations.

Variables	MetS components Beta (*P* value)	MetS components + FPI Beta (*P* value)
Fasting plasma glucose	−0.386 (0.000)	−0.415 (0.000)
Body mass index	0.361 (0.000)	0.269 (0.001)
HDL-C	−0.181 (0.028)	−0.177 (0.017)
Log (FPI)	—	0.288 (0.005)

Beta: standardized coefficients; MetS: metabolic syndrome; HDL-C: high-density lipoprotein cholesterol; FPI: fasting plasma insulin.

## Abbreviations

S_I_: Insulin sensitivity
T2DM: Type 2 diabetes
IR: Insulin resistance
FPIS: First-phase insulin secretion
SPIS: Second-phase insulin secretion
PreDM: Prediabetes
MetS: Metabolic syndrome
BMI: Body mass index
TG: Triglyceride
HDL-C: High-density lipoprotein cholesterol
FPG: Fasting plasma glucose
FPI: Fasting plasma insulin
NGT: Normal glucose tolerance
FSIGT: Frequently sampled intravenous glucose tolerance test
AIRg: Acute insulin response after glucose load
GE: Glucose effectiveness
DI: Disposition index
HOMA-IR: Homeostasis model assessment of insulin resistance
HOMA-*β*: Homeostasis model assessment of the *β*-cell function.
